# How perceived overqualification affects work engagement among new-generation teachers: the mediating role of harmonious work passion and moderating role of humble leadership

**DOI:** 10.3389/fpsyg.2026.1911357

**Published:** 2026-09-03

**Authors:** Li Jiang, Xiaoli Xie

**Affiliations:** 1 College of Education, Zhejiang Normal University, Jinhua, China; 2 Zhengzhou No. 36 Senior High School, Zhengzhou, China; 3 Xinzhou Vocational and Technical College, Xinzhou, China

**Keywords:** harmonious work passion, humble leadership, new-generation teachers, perceived overqualification, work engagement

## Abstract

This study targets post‑1995 highly‑educated secondary‑school teachers in China. Despite accumulating evidence on perceived overqualification (POQ), few longitudinal studies have unpacked its temporally ordered predictive effects on teacher work engagement within educational contexts. Drawing on Relative Deprivation Theory and Self‑Determination Theory, this study adopts an 18‑month three‑wave time‑lagged longitudinal design and constructs a moderated‑mediation model where harmonious work passion (HWP) serves as the mediator and humble leadership (HL) acts as the boundary condition. Based on valid three‑wave data from 498 teachers, hierarchical regression, 5000‑time bootstrap tests and supplementary sequential path analysis are conducted to test the hypothesized temporal predictive associations. Statistical results indicate that POQ exerts significant negative predictive effects on both HWP and work engagement, HWP fully mediates the POQ‑work engagement linkage, and humble leadership buffers the detrimental POQ‑HWP association; when teachers perceive high‑level humble leadership, POQ’s negative influence on intrinsic work passion is weakened, which further attenuates POQ’s indirect adverse effect transmitted via HWP, and time‑lagged results support unidirectional lagged predictive paths that T1 POQ predicts T2 HWP and T2 HWP predicts T3 work engagement. Theoretically, this research establishes an integrated dual‑theoretical framework, validates HWP’s exclusive full‑mediating role among teacher samples, and introduces humble leadership as a novel contextual moderator for education. Practically, school administrators can implement job enrichment, build multi‑layered career pathways and foster humble leadership among supervisors to mitigate qualification‑mismatch‑triggered losses and sustain stable teacher work engagement. This study extends the boundary conditions of POQ research and provides operable strategies for K12 institutions to retain high‑credential young educators.

## Introduction

Intense competition in the labor market often results in organizations recruiting individuals whose qualifications exceed the requirements of the positions. Such conditions have contributed to a widespread perceived overqualification (POQ) among employees, where individuals possess qualifications—such as higher education, experience, and skills—that surpass what their roles require. According to Global Press data, approximately 47% of employees worldwide feel overqualified, a sentiment that is particularly acute in China, where the figure rises to 84%.

POQ can elicit diverse responses, ranging from negative outcomes including feelings of injustice, emotional distress, and diminished work performance (Cheng et al., 2020; (Luksyte et al., 2011); Allan et al., 2020) to positive behaviors like job remodeling and innovation (Zhang et al., 2016). As new-generation employees, who value meaningful work and experiences, enter the workforce, the impact of POQ on work engagement has become increasingly significant. This demographic’s approach to work is profoundly influenced by their perceptions of their jobs, making them particularly vulnerable to the cognitive and emotional effects of perceived overqualification.

Despite extensive research on POQ, gaps remain in understanding how environmental factors, such as leadership style, can mitigate its adverse effects and enhance positive outcomes. This study aims to bridge this gap by examining the role of harmonious work passion (HWP) and humble leadership (HL) in alleviating the negative impacts of POQ on work engagement among new-generation teachers. By exploring how leadership style influences these educators’ passion and engagement, this research may contribute to the field of teacher education.

## Literature review

The contemporary labor market is characterized by a mismatch between the educational qualifications of employees and the requirements of their job roles. This mismatch, referred to as POQ, has profound implications for employee engagement and organizational outcomes. Mottaz (1984) initially introduced the concept of overeducation, which has since been explored through the lens of both objective and subjective overqualification. Objective overqualification refers to measurable excesses in employee qualifications over job requirements, while subjective POQ is an individual’s personal assessment of this excess (Maynard et al., 2006; Verhaest and Omey, 2006).

POQ specifically affects employee attitudes and behaviors more critically than objective metrics. Early research primarily underscored the negative repercussions of POQ, such as reduced job satisfaction (Wassermann et al., 2017), increased feelings of unfairness (Cheng et al., 2020), and greater occupational stress (Maynard et al., 2006). However, studies have also identified potential positive outcomes, suggesting that POQ can enhance innovative behaviors and performance under certain conditions (Purohit, 2018). Recent research explores the nuanced interplay between POQ and work engagement, mediated and moderated by various workplace and personal factors. For instance, Tomás et al. (2023) investigate how organizational size can exacerbate the negative impacts of POQ on work engagement, proposing that larger organizations may fail to provide the relational and developmental supports necessary for overqualified employees, potentially intensifying their disengagement (Tomás et al., 2023). Conversely, Jin et al. (2025) focus on the Conservation of Resources (COR) theory to frame how perceived service climate can enhance work engagement among employees, with POQ serving both as a resource that empowers employees and as a moderating variable that intensifies the beneficial effects of a supportive service climate on engagement. They argue that POQ, by providing a surplus of skills and knowledge, enables employees to leverage organizational resources more effectively, enhancing both in-role and extra-role performance through increased engagement (Jin et al., 2025). Therefore, the research underscores the complexity of POQ’s impact on work engagement, suggesting that the contextual factors within organizations play critical roles in determining whether POQ translates into enhanced engagement or increased disengagement.

Central to the dynamics of POQ is the role of HWP, which acts as a mediating variable in the relationship between POQ and work engagement. HWP, as defined by Vallerand et al. (2003), involves a voluntary and enjoyable immersion in work activities, contrasting with obsessive passion driven by external pressures. This form of passion implies a high degree of integration of work into one’s identity, fostering positive attitudes and behaviors such as increased satisfaction and organizational citizenship behaviors (Forest et al., 2011). Moreover, the role of leadership is crucial in moderating the effects of POQ. Transformational leadership impacts POQ primarily through the mediation of career growth opportunities (Zhang et al., 2021). This leadership style, which emphasizes vision, inspiration, and the personal and professional growth of employees, can mitigate feelings of overqualification by enhancing career development prospects (Liu et al., 2024). This effect is partly due to transformational leaders fostering an environment where employees are encouraged to use their excess skills and competencies, thus aligning their actual job roles with their qualifications and reducing the mismatch that contributes to POQ. Additionally, the positive relationship between transformational leadership and career growth opportunities is strengthened when the level of supervisor–subordinate guanxi (personal connections) is high, indicating that social bonds within the workplace further enhance the beneficial effects of transformational leadership on POQ. While HL, which is characterized by an acknowledgment of one’s limitations, an appreciation of others’ strengths, and openness to feedback, impacts POQ by fostering an environment of work meaningfulness and proactive socialization (Wu et al., 2024). HL enhances work meaningfulness, which in turn encourages employees to perceive their roles as valuable and significant, reducing feelings of overqualification (Wu et al., 2024). HL, characterized by self-awareness, appreciation of others’ strengths, and openness to new ideas (Owens et al., 2013), can mitigate the negative effects of POQ on HWP and by extension, on work engagement. Research has shown that HL enhances employee psychological freedom, thereby fostering a supportive work environment that can counterbalance the negative impacts of overqualification (Nielsen et al., 2010; Owens et al., 2013).

Given the complexities observed in the relationship between POQ and work engagement, limited research has delved into the role of HL as well as HWP within this context. The present study aims to address this gap by exploring how HL and HWP influence the intricate dynamics between POQ and work engagement.

Existing research has identified multiple motivational mediators linking POQ and work engagement, including psychological empowerment, organizational commitment, work meaningfulness, teacher self-efficacy and psychological capital. Nevertheless, this study selects harmonious work passion (HWP) as the exclusive mediator for three theoretical reasons. First, the Dualistic Model of Passion distinguishes harmonious passion from obsessive passion; HWP represents autonomous intrinsic motivation, which highly matches the three basic psychological needs (autonomy, competence, relatedness) proposed by Self-Determination Theory. Other mediators such as organizational commitment focus on organizational attachment rather than spontaneous work enthusiasm. Second, relative deprivation triggered by POQ generates internal emotional dissonance; harmonious work passion directly reflects individuals’ internal affective immersion in work, serving as the proximal emotional bridge between qualification mismatch and engagement loss. By contrast, psychological capital and self-efficacy belong to stable trait variables with weak situational responses to perceived overqualification. Third, existing studies targeting overqualified teachers rarely adopt passion as the mediating mechanism; most research only discusses organizational attitude variables. This study fills the literature gap by revealing how skill mismatch erodes young teachers’ spontaneous work passion, a neglected micro-emotional channel in educational management research.

## Theoretical basis and research assumptions

### Relative Deprivation Theory

Relative Deprivation Theory refers to the objective situation in which an individual is unable to fully predict feelings and behavior, and the subjective evaluation of the situation will also affect perception and behavior, such as the state in which an individual compares his current state with the past or potential state, and the state comparing gains and losses with others. Through comparison, when individuals find a gap between what they get in real life and what they expect to get, they will have some negative emotions caused by deprivation, such as anger, dissatisfaction, depression, etc. (Crosby, 1984). The bigger the difference between employees through comparison is, the stronger their sense of their negative reaction is (Erdogan and Bauer, 2009). When the teachers of the post 1995 generation have improved their abilities through experience accumulation, training and education, they will set their expectations based on their self-evaluation of their qualifications, and judge whether their expectations are met by comparing with colleagues of similar types of work or with their previous work (Erdogan and Bauer, 2009). When working conditions such as challenges, complexity and development opportunities are far below expectations, employees will feel a sense of deprivation, which will lead to negative attitudes and behavior (Liu and Wang, 2012).

### Self determination theory

Self determination theory is a process research theory focusing on people’s self determination behavior motivation, emphasizing that psychological quality plays an important role in self behavior management and personality development (Deci and Ryan, 2000). Self determination theory thinks that there are three types of basic human needs (relational needs, autonomous needs, and competency needs). Based on these three needs, individual motivation can also be divided into three categories: unmotivated, intrinsic motivation, and extrinsic motivation. The theory believes that the way to promote the internalization of external motivation and enhance people’s internal motivation can meet three basic needs of people. Self determination theory explains how extrinsic motivation changes into intrinsic motivation through moderation, and believes that there are many factors that can affect individual motivation, including external environmental factors and personal internal factors. When the post 1995 generation teacher’s POQ triggers negative attitudes and behavior, if there is an environment conducive to meeting their basic needs, such as organizational support (Liu et al., 2015), the negative impact of POQ may be mitigated.

### Research assumptions

According to Relative Deprivation Theory, the objective situation of an individual cannot fully predict his feelings and behavior, and the subjective evaluation generated by the comparison with the past and others will have an important impact on his cognition and behavior. When there is no strong gap between individual expectations and reality, individuals tend to show positive attitudes and behavior. Otherwise, it will stop developing the relationship. Through the evaluation of their own work ability, experience, skills and other aspects, employees can get the expected return value, including the matching of work content and certain growth space. However, when individuals compare reality and find that there is no hope of getting the expected return and producing POQ, they may further evolve into a sense of deprivation, so that individuals are more inclined to stop establishing and developing relationships with work, and avoid excessive loss of their own interests. Similar conclusions have been drawn from previous studies, which suggest that POQ will reduce employees’ work engagement (Kiazad et al., 2015), and even generate turnover intention (Maynard et al., 2006). Therefore, we propose hypothesis 1:

*H1: Perceived overqualification has a negative impact on work engagement.*

According to Relative Deprivation Theory, when the individuals with POQ find that they cannot match the job, has no room for development, and it is difficult to adjust and change the work content and role, they will feel depressed and negative, so that they cannot obtain work meaning and feel work fun spontaneously, which makes it difficult to produce a HWP. Specifically, when employees think that their situation in all aspects exceeds the job requirements, they will have a low sense of self-efficacy (Maynard and Parfyonova, 2013). At the same time, the new generation of teachers with POQ feel that they lack opportunities for growth and learning in the organization, which will aggravate the gap between expectations and reality, and cause them to have negative emotions such as disappointment, which makes it difficult to produce a HWP. Therefore, we propose hypothesis 2:

*H2: POQ has a negative impact on HWP.*

Due to the negative impact of POQ on HWP, at the same time, HWP also has a certain predictive effect on individual behavior. Therefore, we propose the hypothesis that the HWP can mediate the relationship between POQ and work engagement. Specifically, it can be demonstrated from two aspects. Based on the Relative Deprivation Theory, POQ will reduce HWP. Due to the mismatch, the POQ causes individuals to feel that the existing work situation cannot meet their personal qualification level, and individuals cannot adjust their work content and role, thus losing the fun and significance of work. Due to the lack of development, POQ leads teachers to feel that they lack opportunities for growth and learning, which aggravates the gap between reality and expectation, resulting in psychological gap and negative emotions, and reduces harmonious work passion. The lack of harmonious work passion means that individuals cannot see the resources and rewards which are worth exchanging provided by the organization, the attraction of work to individuals is reduced, and individuals lack internal driving force, which leads to individuals tend to stop developing this relationship and reduce the investment in time, energy and other aspects of work. Similar conclusions have been drawn from previous studies (Parker et al., 1997). Therefore, we propose hypothesis 3:

*H3: HWP mediates the relationship between POQ and work engagement.*

According to self determination theory, the internalization of an individual’s external motivation depends on whether the environmental factors can meet the internal psychological needs. HL can reduce the negative impact of POQ on HWP by meeting competency needs, autonomous needs, and relationship needs. From the perspective of competency needs, leaders with high-level HL are more able to recognize the qualifications of employees and conduct an objective and fair inspection of employees, which will reduce the sense of deprivation of employees to a certain extent (Alfes et al., 2016). At the same time, their behavior of modestly consulting and learning from subordinates can make the employees feel that their abilities are recognized by the organization and meet their ability needs. From the perspective of independent needs, leaders with high-level HL will choose to give employees the way of decentralization to full tasks freely and independently, actively provide more opportunities for employees to participate in work decisions, fully respect and recognize the views of subordinates, and provide employees with more satisfaction in terms of work autonomy and influence. From the perspective of relationship needs, leaders with high-level HL will admit personal shortcomings, seek feedback from subordinates, and establish an equal communication mechanism, so that employees have a high sense of respect and interaction fairness (Owens et al., 2013), and strengthen their recognition of leaders and organizations. Therefore, the following assumptions are made:

*H4: Humble leadership buffers the negative association between perceived overqualification and harmonious work passion. Specifically, the negative predictive effect of POQ on harmonious work passion becomes significantly weaker for teachers with high perceptions of humble leadership, while the damaging influence of POQ on HWP is strengthened under low humble leadership.*

*H5: Humble leadership buffers the negative POQ–HWP linkage, further attenuating the negative indirect effect of POQ transmitted via harmonious work passion; the mitigating mediation effect becomes stronger as humble leadership increases.*

**Figure 1:**
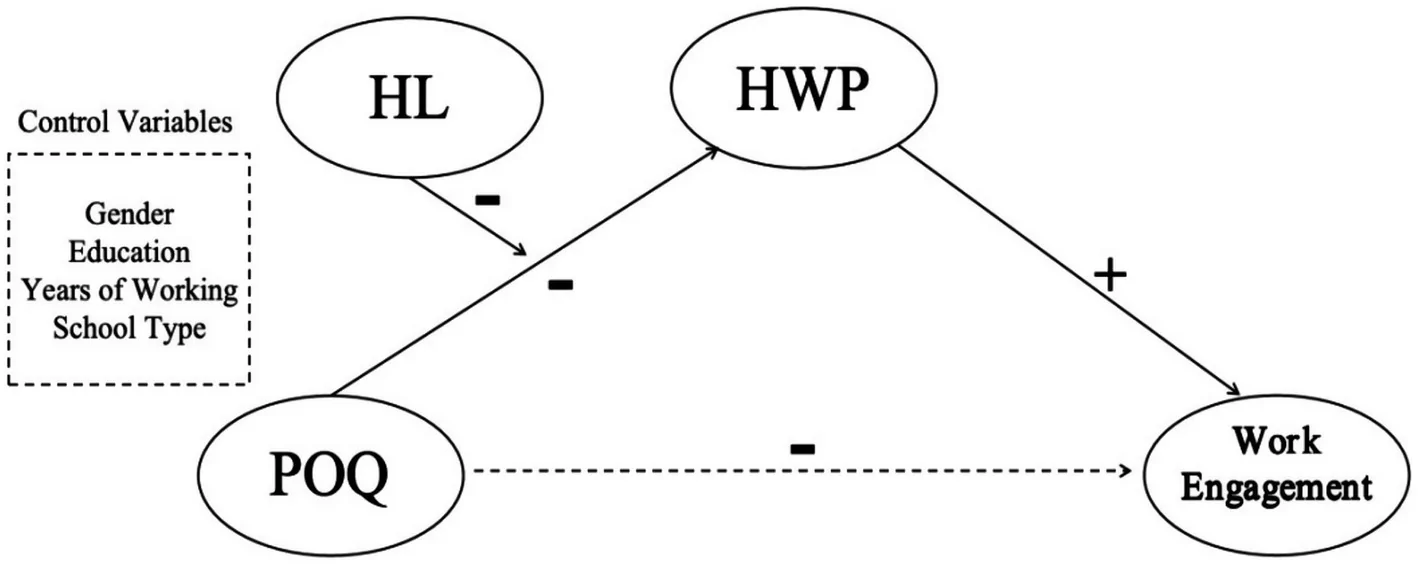
The moderated mediation model of the study.

In summary, this study aims to examine the effects of POQ on work engagement among new-generation teachers, incorporating the mediating role of HWP and the moderating effect of HL. By clarifying how and under what conditions POQ influences work engagement, the research seeks to advance understanding of these mechanisms. Figure 1 presents the proposed moderated mediation model.

## Method

### Measurement

The study employed the SPOQ scale (Maynard et al., 2006) to assess POQ, encompassing aspects such as excess capacity, knowledge, education, and work experience across nine items. This scale is academically validated, demonstrating robust reliability and validity. Work engagement was measured using the revised UWES-9 scale (Schaufeli et al., 2002), available in Chinese and validated in numerous Chinese contexts, which evaluates vitality, dedication, and focus across nine items. HWP was assessed using a seven-item scale developed by Vallerand (2010), and HL was measured with a nine-item scale by Owens et al. (2013), which includes items on valuing others’ contributions and openness to learning.

### Participants

This study employed a cluster sampling method to select 20 secondary schools across three major regions in China, with 12, 6, and 2 schools representing the eastern, central, and western regions, respectively. A total of 1,000 post-1995 generation teachers were recruited from these schools for questionnaire surveys. The overall panel attrition rate reached 50.2% (502 of the initial 1,000 participants). Three practical factors caused heavy sample loss. First, follow-up surveys overlapped with school exams and vacations, leaving young teachers little spare time to complete repeated questionnaires. Second, these elite university graduates have abundant career options; accordingly, many transferred to other jobs or schools and could not be tracked. Third, repeated lengthy surveys triggered response fatigue, so some participants abandoned later waves. To be specific, the sample experienced attrition of 314 participants in the second wave and 188 in the third wave, resulting in a total dropout of 502 individuals. Although demographic comparisons showed no significant gaps between retained and dropped samples, this high attrition creates three methodological risks. For one thing, sample shrinkage lowers statistical power from 0.96 (*N* = 1,000) to 0.83 (*N* = 498), weakening the model’s ability to detect subtle moderated mediation effects. For another, attritors may hold unmeasured negative work attitudes, which restricts the external generalizability of our results. Last but not least, unbalanced panel data reduces the reliability of longitudinal temporal predictive inference based on time-lagged design. To partially offset these limitations and improve the precision of coefficient standard errors and confidence intervals, we applied 5,000-times bootstrap resampling. It is important to clarify that bootstrap resampling cannot eliminate systematic attrition or selection bias caused by panel dropout.

Data were collected in three waves. Wave 1 included the POQ and baseline demographics; Wave 2 (6-month follow-up) assessed HL and HWP; and Wave 3 (12 months post-Wave 2) evaluated work engagement. Ultimately, 498 participants with complete three-wave data were retained for analysis, comprising 242 (48.6%) from the eastern region, 181 (36.3%) from the central region, and 75 (15.1%) from the western region. The sample demonstrated balanced demographic characteristics, with 46.0% male and 54.0% female respondents. The majority (72.7%) held bachelor’s degrees, while the remaining 27.3% possessed master’s degrees. Regarding professional experience, the distribution was as follows: 40.2% had 3–5 years of teaching experience, 19.7% had 1–3 years, 7.2% had less than 1 year, and 33.3% had more than 5 years. Participants were employed in both private (33.1%) and public (66.9%) schools, reflecting the educational attainment and career distribution patterns characteristic of China’s post-1995 generation educators. Importantly, comparative analyses revealed no significant differences between retained and attrited participants in terms of gender [*χ*^2^(1) = 0.05, *p* > 0.05] or work experience [*χ*^2^(5) = 8.69, *p* > 0.05], suggesting that attrition did not systematically bias the sample composition.

### Data collection

Data collection for this study was primarily conducted online via WJX, a safe online survey platform. The research objects are post-1995 generation teachers graduated from top-tier universities, who were selected through stratified cluster sampling covering 20 secondary schools distributed in eastern, central and western China (12 eastern, 6 central, 2 western schools). To ensure the reliability of responses, the survey incorporated three attention-check items (e.g., “I occasionally eat cement”) to filter out inattentive participants. In the WJX platform, only participants who successfully passed all three attention-check questions were retained for subsequent analysis. Prior to participation, all respondents received comprehensive information about the study’s confidentiality measures and provided informed consent in accordance with the ethical guidelines established by the institutional review board and the Declaration of Helsinki (1964) with its subsequent amendments. To mitigate potential order effects and response bias, the survey platform implemented randomized item sequencing for each participant.

## Result

### Bias test

In the design of the questionnaire, measures were implemented to mitigate bias from individual characteristics and environmental influences. Specifically, a verification item was incorporated into the measurement of HWP to ensure respondents’ seriousness. Additionally, the survey’s anonymity was emphasized, assuring confidentiality and no impact on personal outcomes, which aimed to reduce respondent bias.

Post data collection, the Harman single-factor test was applied to assess common method bias across 34 items covering four variables (POQ, HL, HWP, and work engagement). The Kaiser–Meyer–Olkin (KMO) value was 0.940, confirming the appropriateness of factor analysis. As shown in Table 1, the analysis identified six factors with eigenvalues above 1, with the largest factor explaining 39.75% of the variance—below the 50% threshold—indicating no significant common method bias. The cumulative variance explained by the first five factors was 68.17%. These results suggest that the data is free from severe common method bias.

**Table 1:** CMV test (principal component analysis).

Component	Total	Percentage of load	Cumulative percentage
1	13.51	39.75	39.75
2	4.72	13.88	53.63
3	1.63	4.80	58.43
4	1.21	3.57	61.99
5	1.07	3.14	65.13
6	1.03	3.04	68.17

### Reliability analysis

The study conducted reliability analysis on the questionnaire, and tested the internal consistency of four variables. According to the test results, Cronbach’s *α* of four variable measurement scales used in this questionnaire are 0.92, 0.89, 0.87, and 0.89, which are greater than 0.8, indicating that the scale selected in this paper has high stability and reliability, and meets the statistical analysis standard.

### Validity test

The study conducted confirmatory factor analysis on the data to test the discrimination validity among POQ, HL, HWP and work engagement. The test results are shown in Table 2. It can be seen that all indicators of the fitting results of the four factor model meet the requirements, in which *x*^2^/df = 1.86, CFI = 0.94, TLI = 0.92, RMSEA = 0.061, SRMR = 0.05. In contrast, in the three factor model (POQ and HL combined into a single factor), in which *x^2^*/df = 4.14, CFI = 0.77, TLI = 0.71, RMSEA = 0.11, SRMR = 0.14. The two factor model, in which *x^2^*/df = 6.65, CFI = 0.57, TLI = 0.54, RMSEA = 0.15, SRMR = 0.20. The single factor model, in which *x^2^/*df = 6.68, CFI = 0.56, TLI = 0.52, RMSEA = 0.15, SRMR = 0.20.

**Table 2:** Confirmatory factor analysis.

Model	*x* ^2^	df	*x*^2^/df	CFI	TLI	RMSEA	SRMR
Four factor model	962.48	517	1.86	0.94	0.92	0.06	0.05
Three factor model	2154.63	521	4.14	0.77	0.71	0.11	0.14
Two factor model	3486.72	524	6.65	0.57	0.54	0.15	0.20
Single factor model	3512.05	526	6.68	0.56	0.52	0.15	0.20

The four factor model is: POQ, HL, HWP, and work engagement; the three factor model is: POQ + HL, HWP, and work engagement; the two factor model is: POQ + HL + HWP, and work engagement; The single factor model is: POQ + HL + HWP + work engagement.

We further calculated composite reliability (CR) and average variance extracted (AVE) for each latent construct to examine convergent and discriminant validity, with results summarized in Table 3. All Cronbach’s *α* and CR values exceeded the recommended threshold of 0.7, and all AVE values were greater than 0.5, meeting the standard for acceptable convergent validity. To implement the Fornell–Larcker discriminant validity test (Fornell and Larcker, 1981), we extracted the square root of each construct’s AVE and placed these values as diagonal entries in Table 4 for pairwise correlation comparison. This widely adopted psychometric standard ensures each latent construct captures more variance from its own measurement items than it shares with other theoretical variables, ruling out the risk of construct fusion.

**Table 3:** Composite reliability (CR) and average variance extracted (AVE) of latent constructs.

Variable	Numbers	Cronbach’s *α*	CR	AVE	Square root of AVE
Perceived overqualification (POQ)	9	0.91	0.92	0.54	0.73
Humble leadership (HL)	9	0.93	0.93	0.61	0.78
Harmonious work passion (HWP)	7	0.94	0.94	0.66	0.81
Work engagement (WE)	9	0.92	0.93	0.62	0.79

**Table 4:** Correlation matrix, square roots of AVE, and HTMT ratios of all constructs.

Variables	1	2	3	4	5	6	7	8
Gender	–							
Education	−0.10	–						
Working years	0.14^*^	−0.15^*^	–					
School type	−0.28^**^	0.05	−0.14^*^	–				
Perceived overqualification	−0.30^**^	0.05	0.09	0.06	**0.73**	0.31	0.29	0.28
Humble leadership	−0.01	0.07	−0.01	−0.13^*^	−0.30^**^	**0.78**	0.83	0.84
Harmonious work passion	0.03	0.13^*^	−0.03	−0.17	−0.28^**^	0.85^**^	**0.81**	0.84
Work engagement	0.02	0.07	0.08	−0.16	−0.27^**^	0.86^**^	0.93^**^	**0.79**

Values on the bolded diagonal are square roots of AVE; values below the diagonal are Pearson correlation coefficients; values above the diagonal are HTMT ratios. ^*^p < 0.05, ^**^p < 0.01.

We calculated Heterotrait–Monotrait (HTMT) ratios alongside the square roots of average variance extracted to evaluate discriminant validity among latent constructs, with all values organized in Table 4 (Henseler et al., 2015). The four-factor confirmatory factor analysis model shows better fit indices than alternative combined-factor models, while the pairwise correlations between humble leadership, harmonious work passion and work engagement exceed the square roots of their respective AVE values. We interpret the discriminant validity of these three closely connected motivational constructs by integrating model fitting indicators, HTMT ratios and zero-order correlations together.

### Correlation analysis

Pearson correlation analysis was conducted to examine the interrelationships between demographic covariates and core latent constructs, and discriminant validity indicators including HTMT ratios and square roots of AVE were collated in Table 4 for integrated comparison.

The correlation results show that perceived overqualification (POQ) presents significant negative correlations with humble leadership (HL, *r* = −0.30, *p* < 0.01), harmonious work passion (HWP, *r* = −0.28, *p* < 0.01), and work engagement (*r* = −0.27, *p* < 0.01). HL is strongly and positively correlated with HWP (r = 0.85, *p* < 0.01) and work engagement (*r* = 0.86, *p* < 0.01). Meanwhile, an extremely strong positive correlation exists between HWP and work engagement (*r* = 0.93, *p* < 0.01). Multicollinearity diagnosis was further performed, and all variance inflation factors (VIF) ranged from 1.42 to 3.76, far below the critical threshold of 10, which excludes severe multicollinearity concerns in subsequent regression analyses.

To assess discriminant validity, we calculated HTMT ratios alongside the Fornell–Larcker criterion. As shown in Table 4, all square roots of AVE (diagonal entries) exceeded the corresponding inter-construct correlations for POQ with other constructs; however, for the pairs among HL, HWP, and WE—which share a common conceptual grounding in positive motivational psychology—the Fornell–Larcker criterion was not fully satisfied. To further ascertain their distinctiveness, we calculated HTMT ratios (Henseler et al., 2015). All HTMT ratios were below the conservative threshold of 0.85, with the highest being HL–WE (0.84) and HWP–WE (0.84). Moreover, the four-factor CFA model demonstrated significantly better fit than all alternative merged models. Collectively, these results support adequate discriminant validity among the four focal constructs. Overall, the correlation pattern aligns with the theoretical expectations of this research. POQ is negatively linked to teachers’ positive work-related attitudes, while humble leadership correlates positively with both harmonious work passion and work engagement.

### Hypothesis test

#### Test of main effect and mediating effect

The study tests main and mediating effects using hierarchical regression in several steps: (1) Incorporate control variables—education, gender, years of work, and school type; (2) Assess the impact of the independent variable (POQ) on work engagement; (3) Evaluate the influence of POQ on the mediator (HWP); (4) Examine the effect of HWP on work engagement; (5) Analyze the significance of HWP’s impact on work engagement when both POQ and HWP are included. If POQ’s direct influence on work engagement diminishes after adding HWP, then HWP fully mediates this relationship. Conversely, if POQ’s impact remains significant, HWP partially mediates the relationship. These relationships are explored using hierarchical regression with the aforementioned control variables, detailed in Table 5.

**Table 5:** Results of main effect analysis and mediating effect regression analysis.

	Harmonious work passion		Work engagement		Work engagement	
	Harmonious Work Passion Model 1	Harmonious Work Passion Model 2	Work Engagement Model 3	Work Engagement Model 4	Work Engagement Model 5	Work Engagement Model 6
Constant	3.90^***^	4.36^***^	3.74^***^	4.25^***^	−0.21	−0.14
Gender	0.01	−0.13	−0.03	−0.17	−0.03	−0.04
Education	0.20	0.21^*^	0.14	0.15	−0.06	−0.06
Years of working	−0.02	0.01	0.06	0.09	−0.08^***^	0.08
School type	−0.08	−0.08^**^	−0.07	−0.07	0.01	0.01
Perceived overqualification		−0.21^***^		−0.14^***^		−0.02
Harmonious work passion					0.94^***^	0.93^***^
*R* ^2^	0.05	0.14	0.04	0.12	0.87	0.87
Adjusted *R^2^*	0.04	0.12	0.02	0.11	0.87	0.87
△*R*^2^	0.05	0.09	0.04	0.08	0.83	0.00
*F*	3.33^*^	7.65^***^	2.33	6.80^***^	320.05^***^	266.82^***^

^*^p < 0.05, ^**^p < 0.01, ^***^p < 0.001. The coefficient is a non standardized regression coefficient.

Model 4 shows that POQ significantly reduces work engagement (*β* = −0.14, *p* < 0.001), supporting Hypothesis 1. Model 2 results, after adjusting for control variables, indicate that POQ negatively affects HWP (*β* = −0.21, *p* < 0.01), supporting Hypothesis 2. In Model 5, HWP significantly boosts work engagement (*β* = 0.94, *p* < 0.001). Model 6, incorporating both POQ and HWP, confirms HWP’s strong positive impact on work engagement remains (*β* = 0.93, *p* < 0.001), while POQ’s influence becomes non-significant (*β* = −0.02, *p* = 0.33). To establish the mediating role of HWP, hierarchical regression and bootstrap methods with Process 3.5 were used. Mediation analysis, using 5,000 samples, shows HWP significantly mediates between POQ and work engagement [indirect effect = −0.22, 95% CI = (−0.338, −0.099)], confirming Hypothesis 3 (as shown in Table 6). The direct effect’s confidence interval includes zero (−0.057, 0.019), indicating full mediation by HWP.

**Table 6:** The result of mediation effect.

	Effect size	Boot	95% confidence interval	
		SE	Boot 95% CI (Lower)	Boot 95% CI (Upper)
Direct effect	−0.02	0.02	−0.057	0.019
Indirect effect	−0.22	0.06	−0.338	−0.099

#### Moderating effect test

The study explores the moderating effect of HL on the relationship between POQ and HWP using hierarchical regression. The process involves: (1) centralizing POQ and HL data to create an interaction term; (2) constructing Model 1 with HWP as the dependent variable, including control variables; (3) developing Model 2 by adding POQ and HL to the equation; (4) formulating Model 3 by incorporating the interaction between POQ and HL. Results are detailed in Table 7.

**Table 7:** The result of moderating effect (dependent variable: Harmonious Work Passion).

	Harmonious work passion		
	HWP Model 1	HWP Model 2	HWP Model 3
Control variable			
Gender	−0.02	0.01	0.05
Education	0.09	0.03	0.10
Years of working	0.07	0.08	−0.02
School type	0.16	−0.04	−0.03
Main effect			
Perceived overqualification		−0.02	−0.02
Humble leadership		0.85^***^	0.79^***^
Moderating effect			
Perceived overqualification*humble leadership			0.09^**^
*R* ^2^	0.04	0.75	0.76
Adjusted *R^2^*	0.02	0.74	0.75
△*R*^2^	0.02	0.72	0.01
*F*	2.33	119.70^**^	102.17^***^

^*^p < 0.05, ^**^p < 0.01, ^***^p < 0.001. Standardized regression coefficients and two-tailed test.

**Figure 2:**
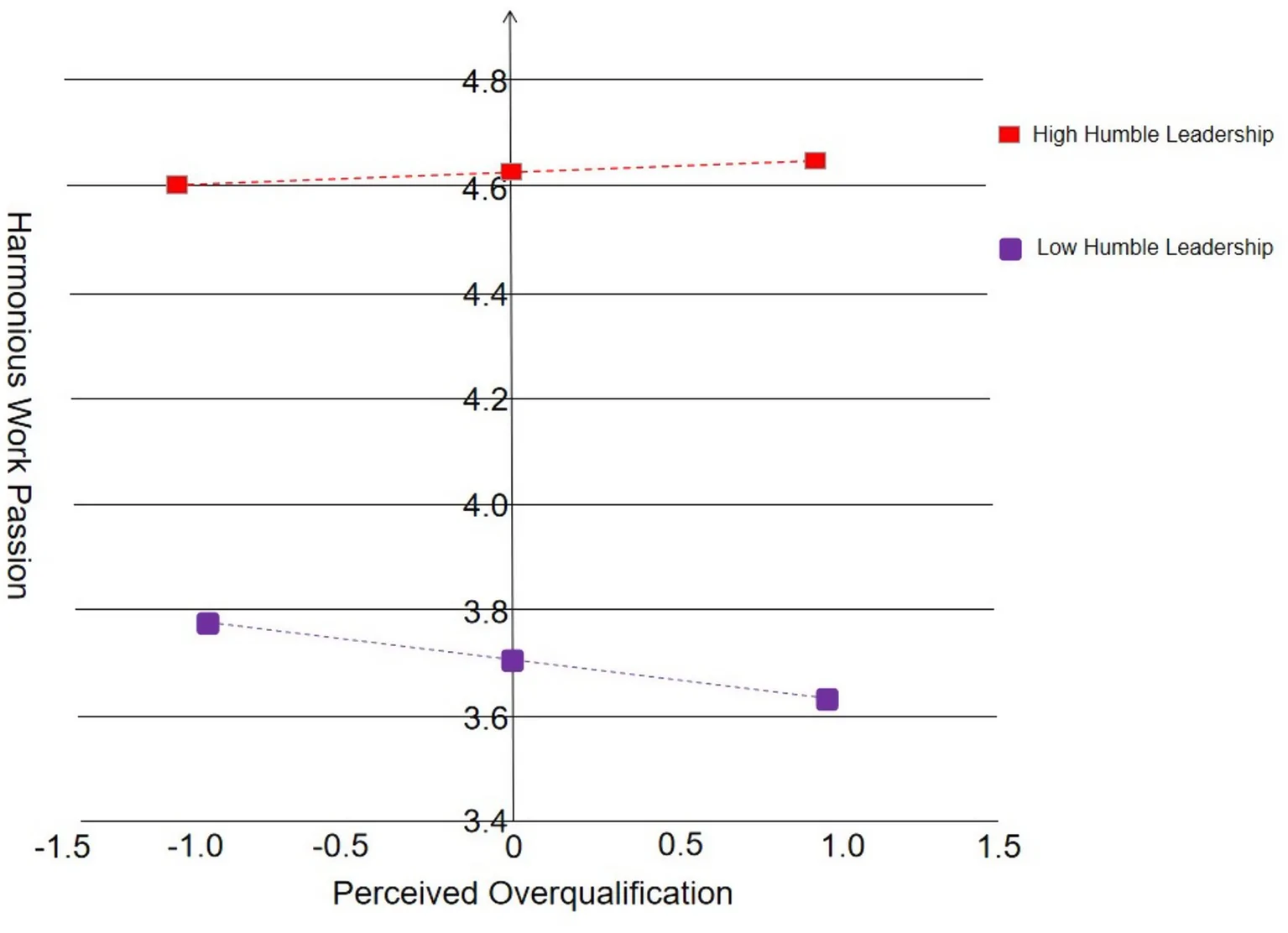
The moderating effect of humble leadership.

In order to more clearly understand the direction and trend of the role of the HL, Process v3.5 plug-in is used to calculate the slope coefficient between POQ, HL, and HWP. As shown in Figure 2, when HL is low, the negative impact of POQ on HWP is enhanced. Therefore, hypothesis 4 was supported, which proposes the buffering moderating effect of humble leadership on the POQ-HWP relationship.

#### Moderated mediation model test

The study utilizes a Bootstrap sampling method to assess a combined mediation and moderation model, with HWP as the mediator and HL as the moderator. To formally test Hypothesis 5 regarding moderated mediation, we computed the index of moderated mediation with 5,000 bootstrap resamples. This index quantifies the magnitude of difference in the indirect effect of POQ on work engagement via HWP at distinct levels of humble leadership. Statistical evidence for moderated mediation is established when the 95% bootstrap confidence interval of this index does not contain zero.

**Figure 3:**
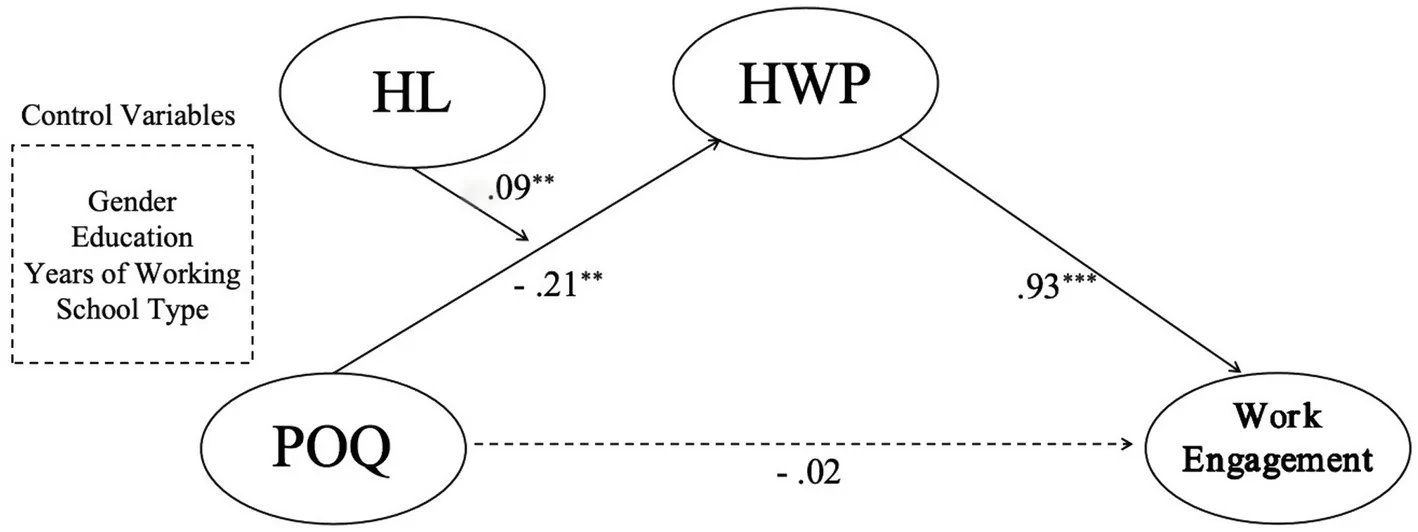
The moderated mediation model of the study.

The results presented in Table 8, Figure 3 support Hypothesis 5. At low levels of HL, the indirect negative effect of POQ on work engagement through HWP was significant [95% CI = (−0.234, −0.015)], meaning weaker humble leadership strengthens the adverse indirect influence of POQ. This conditional indirect effect turned non-significant at medium and high HL levels. We further calculated the index of moderated mediation: the index value was 0.09, bootstrap standard error = 0.041, 95% CI = [0.005, 0.2486]. The confidence interval excludes zero, which provides statistical proof for the moderated mediation effect proposed in Hypothesis 5.

**Table 8:** The influence of different levels of humble leadership on mediating effect.

Humble leadership	Indirect effect	Boot	95% confidence interval	
		SE	Minimum	Maximum
Low	−0.08	0.043	−0.23	−0.02
Middle	−0.02	0.08	−0.09	0.31
High	0.03	0.04	−0.03	0.10

## Three-wave time-lagged sequential path analysis

To examine the hypothesized sequential temporal predictive association based on our three-wave separated measurement design, we established this time-lagged sequential path model. Table 9 reports the estimated forward lagged predictive paths. The paths from T1 POQ to T2 HWP and T2 HWP to T3 work engagement reached statistical significance. This result provides tentative temporal predictive evidence supporting our core mediation chain: perceived overqualification reduces harmonious work passion first, which subsequently lowers teachers’ work engagement 1 year later.

**Table 9:** Sequential time-lagged path coefficients.

Path	Unstandardized *B*	SE	*p*	95% confidence interval
Forward lagged paths				
T1 POQ → T2 HWP	−0.19	0.04	<0.001	[−0.27, −0.11]
T2 HWP → T3 work engagement	0.91	0.03	<0.001	[0.85, 0.97]

## Conclusion

This study, grounded in Relative Deprivation Theory and Self-Determination Theory, explored the effects of POQ on work engagement among 498 middle school teachers from China’s post-1995 generation, who graduated from top universities. It found that POQ negatively impacts work engagement and HWP, with the latter fully mediating the relationship between POQ and work engagement. Moreover, humble leadership buffers the negative POQ–HWP linkage and further attenuates the negative indirect effect of POQ transmitted via harmonious work passion. The three-wave time-lagged sequential path analysis detected significant unidirectional lagged predictive links from T1 POQ to T2 HWP and T2 HWP to T3 work engagement. These temporal patterns align with our hypothesized sequential association. To manage the challenge of overqualified teachers effectively, educational institutions may consider implementing policy changes such as job enrichment programs that engage teachers in complex tasks, clear career development opportunities, flexible role assignments to utilize diverse skills, and mechanisms for recognizing and deploying teachers’ excess skills in areas like curriculum development or peer training. These measures can help reduce the negative impact of overqualification by enhancing job satisfaction and engagement, ultimately contributing to educational success and organizational effectiveness.

### Discussion

The study confirms that POQ directly correlates to lower work engagement, primarily due to overqualified teachers feeling constrained and unable to utilize their full capabilities, leading to negative emotions and behaviors. Additionally, a gap between their expectations and the actual job, as explained by Relative Deprivation Theory, results in decreased engagement and increased job burnout (Luksyte et al., 2011). This aligns with previous findings that higher levels of POQ correlate with reduced work engagement. Further three-wave time-lagged sequential path analysis provides tentative temporal predictive evidence consistent with this cross-sectional correlation: the significant unidirectional lagged paths T1 POQ → T2 harmonious work passion and T2 HWP → T3 work engagement support our hypothesized sequential predictive chain. POQ significantly reduces HWP. According to Self-Determination Theory, overqualification fails to meet psychological needs for competence and integration within teams, thereby diminishing spontaneous enthusiasm for work. This reduction in passion negatively influences job satisfaction and perceptions of fairness, which in turn affects overall morale and engagement (Cheng et al., 2020). HWP strongly enhances work engagement, with high passion leading to greater intrinsic motivation and immersion in work roles. Teachers with high work passion view their work as an integral part of their identity, which fosters a supportive interpersonal network, enhancing workplace contributions and satisfaction (Jowett et al., 2013). Conversely, lower passion leads to behaviors that deviate from organizational goals (Fernet et al., 2014). The relationship between POQ and work engagement is fully mediated by HWP. High POQ creates a psychological gap and negative emotions, which diminish work passion and negatively impact job engagement (Maynard et al., 2013). Additionally, humble leadership significantly moderates the relationship between POQ and HWP. Low levels of HL exacerbate the negative effects of POQ on work engagement through work passion. However, as HL increases, this buffering effect gradually attenuates, rendering the conditional indirect effect non-significant at medium and high levels of HL. This pattern, combined with the significant index of moderated mediation [index = 0.09, 95% CI = (0.005, 0.2486)], confirms that the indirect effect varies significantly across HL levels. The negative indirect effect of POQ on work engagement via HWP is the strongest when humble leadership is low. Higher humble leadership significantly buffers and attenuates this adverse indirect association.

The diminishing marginal buffering benefit of high HL, rather than indicating a “harmful” effect, can be explained by three educational context-based mechanisms. First, role ambiguity mechanism. For highly educated overqualified new-generation teachers, frequent leader humility and repeated acknowledgment of leaders’ limitations may create ambiguous job boundaries, making teachers uncertain whether their extra professional skills can be formally applied to curriculum innovation, which partially offsets the satisfaction of competence psychological needs. Second, status mismatch perception mechanism. Overqualified teachers hold high self-perceived professional status; excessive humble leadership blurs hierarchical guidance boundaries, reduces structured career development support, and weakens the alleviation of relative deprivation. Third, primary and secondary school management specificity. Daily teaching requires standardized management and clear accountability. Over-frequent humble leadership may delay decision-making on job enrichment and teacher training, slowing the resolution of skill-job mismatch (Owens et al., 2013). Though high HL still produces obvious buffering compared with low HL, its marginal mitigation benefit declines at extremely high levels, suggesting that humble leadership functions most effectively as a buffer when it is present at moderate, rather than excessive, levels.

Beyond replicating the widely documented negative POQ-work engagement relationship, this study makes three incremental theoretical contributions to overqualification and teacher motivation literature. First, we construct a dual theoretical framework integrating Relative Deprivation Theory and Self-Determination Theory to explain the complete moderated mediation chain of POQ. Most existing studies only adopt one single theoretical lens; the dual framework simultaneously interprets comparative deprivation from skill-job mismatch and the motivational buffering path shaped by humble leadership, enriching multi-theoretical explanatory perspectives of overqualification harm mechanisms. Second, this study introduces humble leadership as a unique contextual boundary condition for K12 education settings, filling the research gap that few studies explore school principal humble leadership as a moderator in overqualification research. Most prior POQ moderators focus on organizational climate or individual personality traits, lacking educational leadership evidence for highly educated new-generation teachers. Third, we identify harmonious work passion as the full intrinsic motivational mediator in teacher samples, distinguishing autonomous harmonious passion from obsessive passion and clarifying that overqualification impairs engagement through identity-integrated internal motivation rather than general organizational attachment. We contextualize the Dualistic Model of Passion within Chinese secondary education labor mismatch and realize domain-specific theoretical refinement for teacher occupational groups. This study expands on the outcomes of POQ and explores its dual impacts on the cognition and behavior of new-generation teachers. Strategies to mitigate POQ should focus on enhancing internal motivation and integrating environmental adjustments like appropriate HL, which can moderate its negative effects.

### Limitations and future directions

This study has several limitations that suggest directions for future research. First, this study adopts a three-wave time-lagged design where distinct constructs are measured at separate survey waves rather than collecting full indicator data for all latent variables in each round of investigation. This data collection arrangement only allows us to examine unidirectional temporal predictive paths across time points, and cannot support comprehensive examination of reciprocal bidirectional predictive relationships among perceived overqualification, harmonious work passion, humble leadership and work engagement simultaneously. Follow-up longitudinal research can arrange repeated measurement of all core variables at every wave to conduct more rigorous tests on mutual predictive effects between constructs. Second, the substantial sample attrition (50.2%) represents a notable methodological concern. Although demographic comparisons revealed no significant differences between retained and attrited participants, the high dropout rate may compromise statistical power (reduced from 0.96 to 0.83) and limit generalizability, particularly if attritors systematically differed on unmeasured psychological constructs. Unbalanced panel data may also attenuate the reliability of longitudinal temporal predictive inference. To partially mitigate these concerns, we employed 5,000-times bootstrap resampling. Future research should adopt shorter survey intervals or incentive strategies to improve retention. Third, while the findings suggest that excessive humble leadership weakens its buffering effect on POQ, the threshold for “excessive” remains unclear, requiring further theoretical and operational refinement. Finally, reliance on self-reported data may introduce bias. Future research should incorporate multi-source or objective measures to strengthen validity.

## Data Availability

The original contributions presented in the study are included in the article/supplementary material, further inquiries can be directed to the corresponding author.
